# Evaluation of DiaSorin Liaison^®^ calprotectin fecal assay adapted for pleural effusion

**DOI:** 10.1515/almed-2023-0106

**Published:** 2023-11-14

**Authors:** Cristina de Paz Poves, Clara Barneo-Caragol, Ana Isabel Cillero Sánchez, Lucía Jimenez Mendiguchia, Covadonga Quirós Caso, María Moreno Rodríguez, Francisco J. López González, Mᵃ Belén Prieto García

**Affiliations:** Laboratory of Medicine, Department of Clinical Biochemistry, Hospital Universitario Central de Asturias, Oviedo, Asturias, Spain; Department of Clinical Biochemistry, Hospital Álvarez Buylla, Mieres, Asturias, Spain; Department of Clinical Biochemistry, Hospital Universitario San Agustín, Avilés, Asturias, Spain; Department of Pneumology, Hospital Universitario Central de Asturias, Oviedo, Asturias, Spain

**Keywords:** automation, calprotectin, chemiluminescence, pleural fluid, validation

## Abstract

**Objectives:**

Calprotectin (CP) is a calcium and zinc binding protein that is widely measured on faecal samples but its determination in other biological fluids might be of interest. The aim of this work was to validate the measurement of CP in pleural fluid by chemiluminescence.

**Methods:**

LIAISON^®^XL, a fully automated chemiluminescence analyzer, was used for CP quantification on pleural fluid. A validation protocol was designed using both quality control materials provided by the manufacturer and pools of pleural fluid samples. Stability, imprecision, bias, linearity, detection capability and carry over effect were evaluated.

**Results:**

CP was stable on pleural fluid at least one week, under refrigerated conditions, and four weeks at −80 °C. The observed intra- and inter-day imprecision was between 2.2 and 6.49 %, with a negative bias under 5.51 %. The linearity of the method was verified up to 2,000 ng/mL. The LoQ for the assay was 48.52 ng/mL. A statistically significant carry-over effect was observed after measuring CP concentrations above the upper limit of linearity, but given the observed magnitude, a clinically relevant impact should not be expected.

**Conclusions:**

DiaSorin Liaison^®^ calprotectin assay allows reliable measurement of CP in pleural fluid.

## Introduction

Calprotectin (CP) (S100A8/S100A9) is a calcium and zinc binding protein of 36 kDa, member of the S100 family of proteins [1]. It is mainly released from the neutrophils, with high levels described in inflammatory processes as a sign of the neutrophil chemotaxis [2].

In clinical practice, fecal CP measurement [3] is widely used for the diagnosis and monitoring of patients suffering from chronic inflammatory gastrointestinal disorders [4], but CP can be measured in other biological fluids [5], such as pleural effusions. Recent studies have pointed out its clinical utility to accurately predict malignancy, but all have used ELISA procedures [6], [7], [8] which, among other limitations, are not suitable for tests that require short response times.

However, fully automated, fast and reliable methods are nowadays available. The aim of this work was to validate the measurement of CP in pleural fluid by chemiluminescence, hypothesizing that this method could provide better clinical performance than ELISA.

## Materials and methods

### Assay principle and instrument

LIAISON^®^XL (DiaSorin, Italy), a fully automated chemiluminescence analyzer, was used for CP quantification on pleural fluid. This analyzer was designed for the quantification of fecal calprotectin so results are expressed in µg/g. Based on previous publication [9] a conversion factor of 2.5 was used to transform these units into ng/mL for the pleural fluid sample. The calibration curve was also transformed, being the measurement range of 12.50–2,000 ng/mL.

### Materials

A validation protocol was designed using quality control materials provided by the manufacturer (Control 1 and 2) and pools of pleural fluid samples.

Twenty pleural fluid samples obtained by thoracentesis were collected on sodium heparin tubes, immediately sent to the laboratory and centrifuged for 5 min at 415×*g*. Then, the supernatant was separated to be analyzed.

Pools were made by homogeneous mixing of various pleural fluid samples until reaching the desired concentration.

### Stability

Stability was evaluated following the Guidelines from Spanish Society of Laboratory Medicine (SEQC^ML^) [10].

First, three sample pools with different CP concentrations were obtained from nine pleural fluid samples (three samples each pool): pool 1 (350 ng/mL), pool 2 (11,340 ng/mL), and pool 3 (145,525 ng/mL). All the pools were split into different aliquots. Three aliquots of each concentration were stored at 4 °C and five at −80°. Refrigerated aliquots were analyzed every 2–3 days over a week, whereas frozen aliquots were analyzed once a week over 5 weeks. Stability was expressed as the difference between concentration at each storage condition and its correspondent basal level (percentage deviation, PD %). Maximum permissible instability (MPI) was set at ±10 %.

### Imprecision and bias

Imprecision was evaluated according to Clinical and Laboratory Standards Institute (CLSI) guideline EP15 [11]. Two pools obtained from six pleural fluid samples (three samples each pool) with concentrations similar to quality control materials were tested, CP target value being 607.5 ng/mL and 121.12 ng/mL for Controls 1 and 2, respectively. Five replicates were run on a single day (intra-day imprecision) for five consecutive days (inter-day imprecision). The referred two levels of quality controls were also tested in three replicates for five consecutive days. Results were expressed as % coefficient of variation (CV).

Bias was estimated based on internal quality control materials.

### Linearity

The guideline CLSI EP06 [12] was followed to verify the linearity. A high concentration pool obtained from three pleural fluid samples (−10 % of upper quantification limit) was diluted with a low concentration pool obtained from another three pleural fluid samples (+10 % of lower quantification limit) in different proportions (1, 0.75, 0.5, 0.25 and 0 % of high pool), covering the dynamic range of the assay. Duplicates of each level were run on a single day. Linearity was assessed by plotting the % deviation between measured vs. predicted values. The allowable deviation from linearity (ADL) was set at ± <bias.

### Detection capability

The claimed blank (LoB), detection (LoD) and quantification (LoQ) limits (0.267, 0.9875 and 12.50 ng/mL, respectively) were studied by following the CLSI EP17 [13] document. Commercial diluent (“blank samples”) as well as eight pleural fluid samples with very low CP concentrations, close to the LoD (“detection samples”) or to the LoQ (“quantification samples”) indicated by the manufacturer, were used for this purpose.

LoB was calculated with a non-parametric test by calculating 95th percentile for the distribution of blank sample results. LoD was calculated as: LoB+Cp×standard deviation (SD) across detection sample results, where Cp is a multiplier to give the 95th percentile of a normal distribution. LoQ was determined as the measurand concentration at the intersection of a power function model fit line (within-laboratory precision (as %CV) vs. quantification samples results) with the accuracy goal of a 10 %CV.

### Carry-over effect

Based on recommendation 1991 IUPAC [14], carry-over effect was evaluated by measuring two pleural fluid sample pools obtained from three pleural fluid samples with high (a) and three with low (b) concentrations of CP. A sequence of two successive aliquots of pool with a high value (a) followed by a sequence of three successive aliquots with low concentration (b) was used. This sequence (a1, a2, b1, b2, b3) was processed for 10 times on a single run.

### Statistical analysis

Statistical analyses were performed with MedCalc^®^ (12.5.0) and Excel (16.70). Continuous variables were described as mean and SD, whereas discrete variables were expressed as number or percentages. Normal distribution was evaluated using the Kolmogorov–Smirnov test. A p-value lower than 0.05 was considered statistically significant. Wilcoxon signed rank test was used for comparison between groups of paired samples.

### Ethics

This study complies with the World Medical Association Declaration of Helsinki regarding ethical conduct of research involving human subjects. Informed consent was obtained from all participants, under a protocol approved by the Authonomic Research Ethics Committee (code number 2022.245).

## Results

### Stability

**Figure 1:**
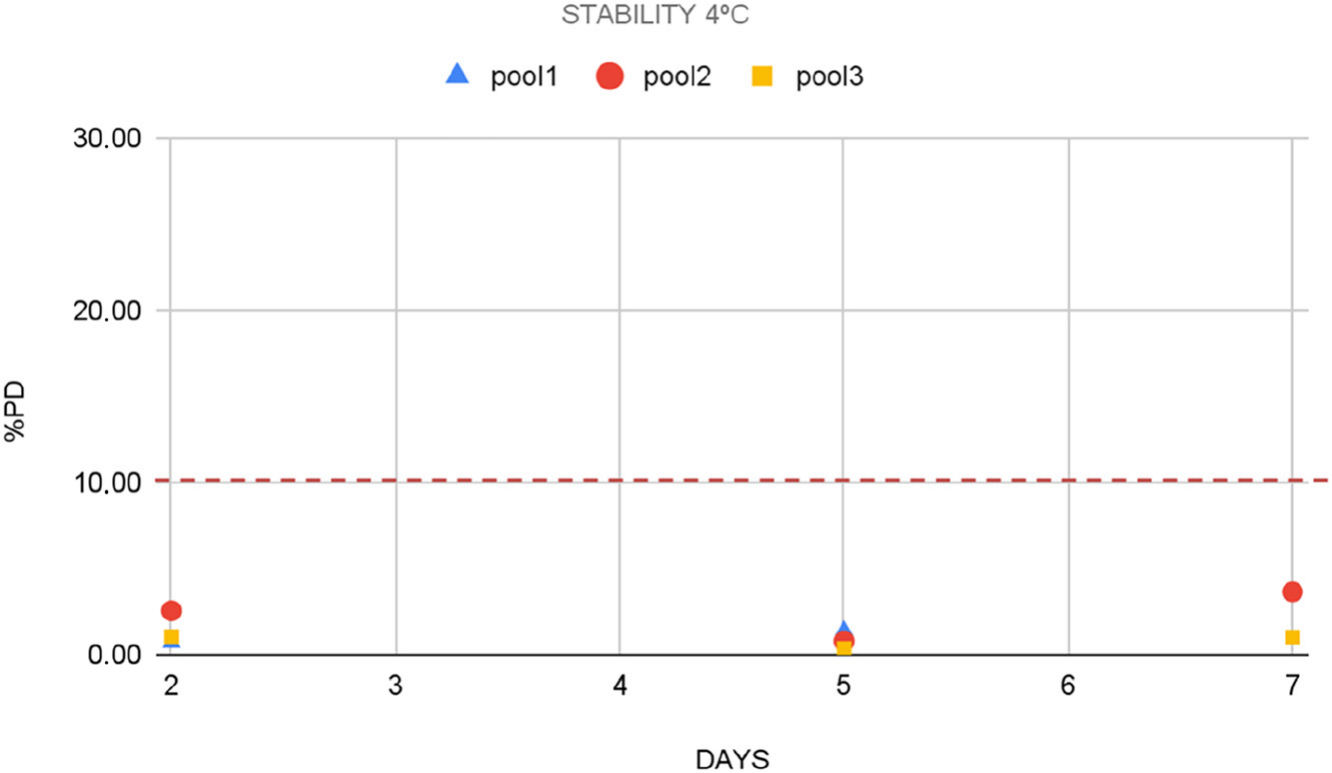
Stability of pools 1, 2 and 3 stored at 4 °C expressed as PD (%). PD, percentage deviation.

**Figure 2:**
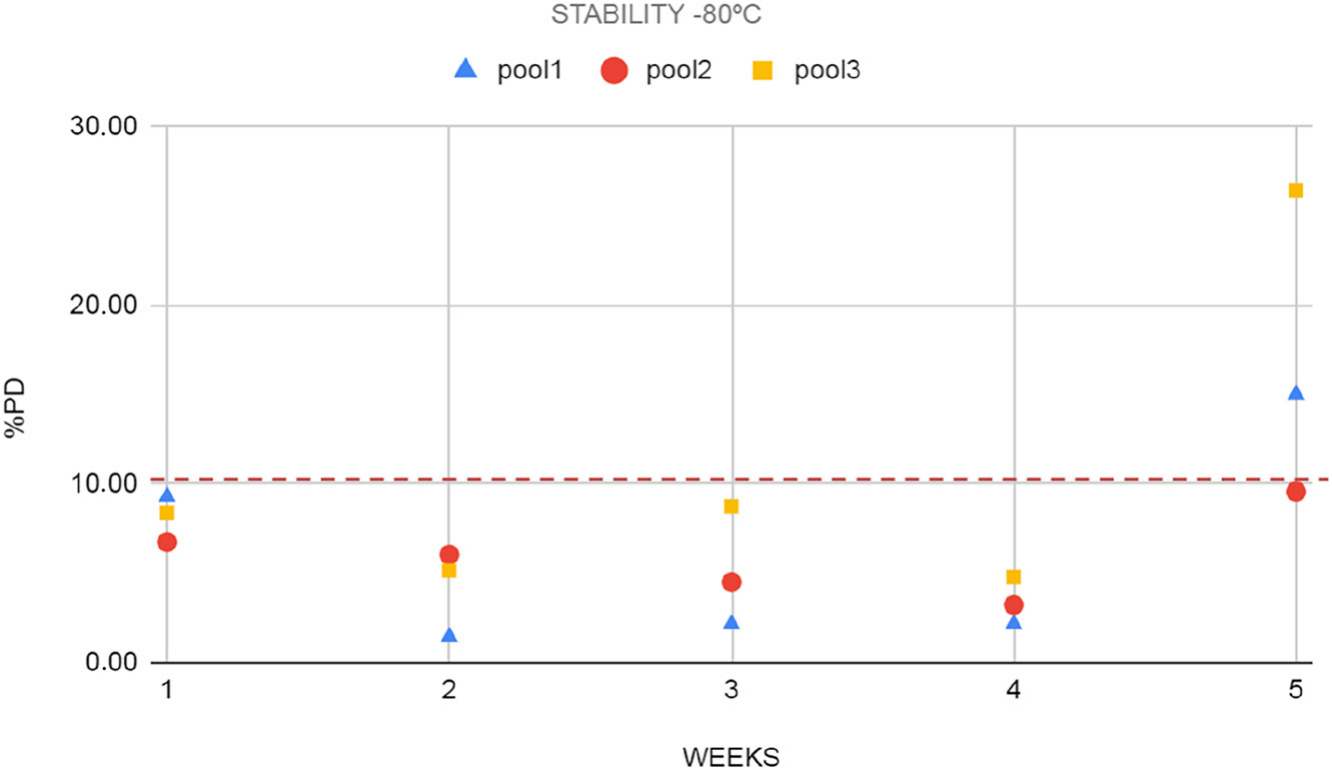
Stability of pools 1, 2 and 3 stored at −80 °C expressed as PD (%). PD, percentage deviation.

CP was stable on pleural fluid at least one week, under refrigerated conditions (Figure 1), and four weeks at −80 °C (Figure 2). At both temperatures, percentage deviation was lower than the maximum permissible instability (MPI) (10 %).

### Imprecision and bias

The observed intra- and inter-day imprecision were similar in the internal quality control materials than in pleural fluid sample pools, ranging between 2.2 and 6.5 %. A negative bias of 3.0 and 5.5 % was also estimated (Table1).

**Table 1:** Study of imprecision (intra and inter-day) and bias.

	Measured CP, ng/mL	Intra-day CV, %	Inter-day CV, %	Bias, %
High level pleural fluid pool	697.5	5.0	6.5	–
Low level pleural fluid pool	153.2	4.7	5.5	–
Control 1 (target 607.50 ng/mL)	574	4.3	4.0	−5.5
Control 2 (target 121.12 ng/mL)	117.5	2.2	5.9	−3.0

CV, coefficient of variation; CP, calprotectin.

### Linearity

**Figure 3:**
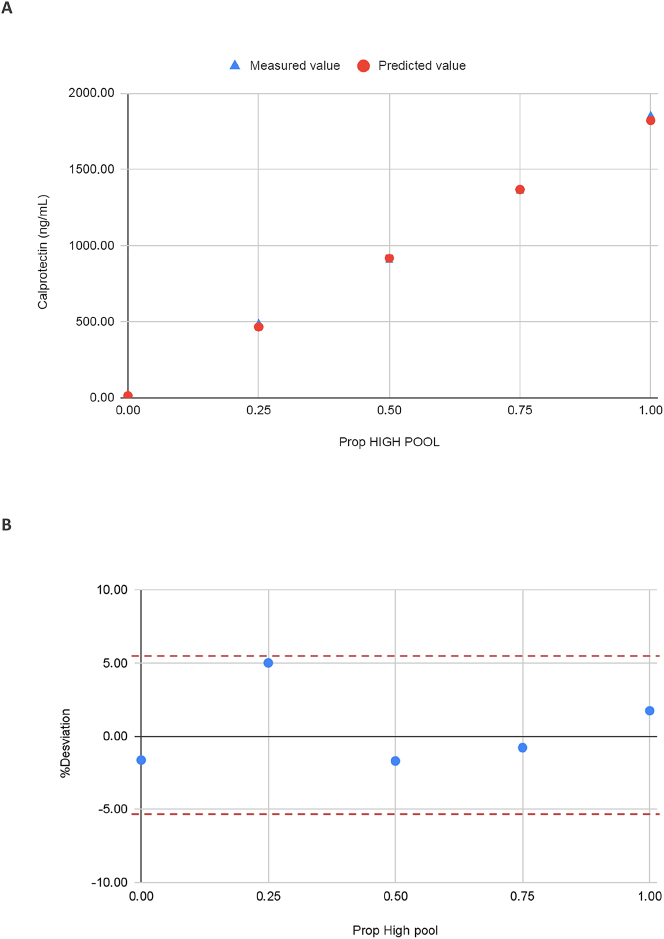
DiaSorin Liaison^®^ calprotectin assay linearity study. (a) Predicted vs. measured values. (b) %Deviation between measured value vs. predicted value according to the proportion of high pool.

The linearity of the method was verified from 12.5 up to 2,000 ng/mL (Figure 3). Over the entire measurement range, the observed ADLs were below the allowable bias.

### Detection capability

The calculated LoQ for the assay was 48.52 ng/mL. Since linearity less than 12.50 ng/mL (6,453 chemiluminescence units, RLU) was not demonstrated, LoB and LoD estimates could not be reported, both being approximately 4 times lower than the LoQ.

### Carry over effect

Median CP levels in the b1 group were significantly higher than in the b3 group [354 (348–360) vs. 339 (332–345) ng/mL, p<0.05]. The experiment was repeated with lower values of (a) close to the upper limit of the linear range, obtaining [329 (315–330) vs. 316 (315–325) ng/mL, p<0.05] (Table 2). A statistically significant mean carryover effect of 3.62 and 2.92 % was observed respectively.

**Table 2:** Carry-over study results observed in two scenarios: very high CP levels (up), and close to the upper limit of the linearity levels (down).

	a1	a2	b1	b2	b3	Carry over effect (p-value)
Very high pool CP median, ng/mL	10,912	10,962	354	347	338	0.0488
High pool CP median, ng/mL	2,145	2,244	329	320	316	0.0195

## Discussion

Currently, in clinical practice, CP levels are routinely measured just in fecal samples in the diagnosis and follow-up of patients suffering from chronic inflammatory gastrointestinal disorders [4].

However, CP has been postulated in recent years as an emerging biomarker to monitor the follow-up of active disease in other autoimmune disorders, since CP plays a pivotal role in innate immunity [15]. In fact, several examples of its clinical utility have already being described, such as the finding of high CP levels in plasma and serum from rheumatoid arthritis (RA) [16, 17] and axial spondyloarthritis (axSpA) [18] patients.

CP also can be found in other biological fluids as effusions: it seems that in synovial fluid CP measure has interest in RA patients [16, 17] and in ascitic fluid for diagnosis of spontaneous bacterial peritonitis in patients with liver cirrhosis [19], [20], [21]. With regard to pleural fluid, CP is involved in numerous cellular processes in lung health and disease. In addition to its antimicrobial functions, CP is also a molecule with pro- and anti-tumor properties related to cell survival and growth, angiogenesis, DNA damage response, and extracellular matrix remodeling [22]. Recent evidence reports its usefulness in predicting malignancy [6], [7], [8] and differentiating between parapneumonic and non-parapneumonic pleural effusions [22, 23]. Nevertheless, studies examining CP levels in pleural effusions are very scarce and have limitations of the measurement techniques used, since they were based on ELISA procedures, despite that, in recent years, fully automated analyzers have been developed. In this study, the analytical performance of the CP measurement on pleural fluid, by a fully automated chemiluminescence assay (DiaSorin Liaison^®^), has been described for the first time.

In the present work, we have demonstrated that the stability of CP in pleural fluid (7 days) is even higher than in feces (72 h), and in EDTA plasma samples (4 days) [24] under refrigerated conditions.

The observed imprecision in pleural fluid sample pools is lower than that described by ELISA method (≤8.67 %, ≤12.82 %, intra- and inter-day, respectively) [6], similar to that found in our internal quality control materials, and slightly higher than the claimed by the manufacturer for stool samples (≤3.10 %, ≤4.00 %, intra-and inter-day, respectively). A negative bias was observed in the internal quality control materials within the margin of the method imprecision. Therefore, this control material could be used in future studies in which pleural fluid is used as a matrix, considering introducing this matrix in the manufacturer’s insert.

The claimed linearity has been verified in LP.

A slightly higher LoQ was observed in pleural fluid than in feces, that should be taken into consideration for future applications where low CP concentrations have clinical interest. Even so, LoQ by this method is lower than that described by ELISA (ELISA fCAL^®^) or lateral flow immunocromatographic method (Quantum Blue^®^) (400 and 5,000 ng/mL respectively) [8].

Finally, a slight carryover effect can be observed not only when measuring very high CP concentrations, but also near the upper limit of the linearity range. However, being within the tolerance margins of analytical performance, clinically relevant differences due to carryover should not be expected.

In conclusion, this study demonstrates that automated chemiluminescence assay provides better clinical performance than other methods described in previous studies.

## Conclusions

DiaSorin Liaison^®^ calprotectin assay allows reliable measurement of CP in pleural fluid and the analytical performance described here encourages further evaluation of its potential usefulness in clinical practice.
